# Activation of Neck and Low-Back Muscles Is Reduced with the Use of a Neck Balance System Together with a Lumbar Support in Urban Drivers

**DOI:** 10.1371/journal.pone.0141031

**Published:** 2015-10-16

**Authors:** Federica Menotti, Luciana Labanca, Luca Laudani, Arrigo Giombini, Fabio Pigozzi, Andrea Macaluso

**Affiliations:** 1 Department of Movement, Human and Health Sciences, University of Rome Foro Italico, Rome, Italy; 2 Department of Medicine and Health Sciences, University of Molise, Campobasso, Italy; Universite de Nantes, FRANCE

## Abstract

Driving is associated with high activation of low-back and neck muscles due to the sitting position and perturbations imposed by the vehicle. The aim of this study was to investigate the use of a neck balance system together with a lumbar support on the activation of low-back and neck muscles during driving. Twelve healthy male subjects (age 32±6.71 years) were asked to drive in two conditions: 1) with devices; 2) without devices. During vehicle accelerations and decelerations root mean square (RMS) of surface electromyography (sEMG) was recorded from the erector spinae, semispinalis capitis and sternocleidomastoid muscles and expressed as a percentage of maximal voluntary contraction (MVC). The pitch of the head was obtained by means of an inertial sensor placed on the subjects’ head. A visual analog scale (VAS) was used to assess the level of perceived comfort. RMS of the low back muscles was lower with than without devices during both acceleration and deceleration of the vehicle (1.40±0.93% vs 29 2.32±1.90% and 1.88±1.45% vs 2.91±2.33%, respectively), while RMS of neck extensor muscles was reduced only during acceleration (5.18±1.96% vs 5.91±2.16%). There were no differences between the two conditions in RMS of neck flexor muscles, the pitch of the head and the VAS score. The use of these two ergonomic devices is therefore effective in reducing the activation of low-back and neck muscles during driving with no changes in the level of perceived comfort, which is likely due to rebalancing weight on the neck and giving a neutral position to lumbar segments.

## Introduction

The driving sitting position is featured by non-neutral spinal postures, generally a reduction in natural lumbar lordosis [1] and an increased or decreased neck flexion [2]. As a consequence, a number of adaptations in muscle activation occur [2,3]. Early studies on sitting posture have reported an increase in activation of low back muscles in absence of lumbar support or back rest [3]. Furthermore, the activation of neck muscles is influenced by the position of other spinal segments, for example an increased lumbar flexion is related to a higher neck activation of the extensors muscles [2]. Altered activations of both low-back and neck muscles during driving may be ascribed to two main factors: first, drivers maintain the same sitting position for a long time; second, they need to adequately control posture during the continuous changes in velocity of the vehicle, i.e. accelerations and decelerations [4,5].

There have been a number of studies looking at the effectiveness of ergonomic devices to reduce driving-related back disorders [6,7]. Lumbar support reduces activation of low back muscles [6] and limits low back pain while driving [7]. To the best of the authors’ knowledge, there are no studies looking at the effectiveness of ergonomic devices for driving-related neck disorders. However, Pavan et al. [8] and Giombini et al. [9] have recently demonstrated that a cap which includes a padding mass over the occipital region, referred to as neck balance system, was able to improve cervical posture and reduce neck pain during activities of daily living. The assumption was that the mass applied over the occipital region could reduce neck extensor activation without increasing flexor muscles activity by rebalancing head weight distribution on the neck. As an increased lumbar flexion is related to a higher neck activation of the extensors muscles [2], using a lumbar support together with a neck balance system may be effective in reducing the activation of both neck-extensor and low-back muscles during accelerations and decelerations of a vehicle.

The aim of this study was to investigate the use of a neck balance system together with a lumbar support for both cervical and lumbar rebalance on the activation of low-back and neck muscles during driving in an urban contest. The hypothesis is that the devices used together will lower the load on these structures by reducing activation of both the neck-extensor and low-back muscles during the changes in velocity of the vehicle, rebalancing weight on the neck and giving a neutral position to the lumbar spinal segments.

## Materials and Methods

### Participants

Twelve healthy male subjects (mean age 32 ± 6.71 years, mean body mass 79.9 ± 6.5 kg) participated in the study. Volunteers were recruited according to the following inclusion criteria: 1) age between 25 and 45 years; 2) no symptoms of low back and cervical pain.

The study was approved by the Ethics Committee of the University of Rome La Sapienza and written informed consent was obtained from all volunteers before the onset of the experimental procedures. The individuals in this manuscript have given written informed consent (as outlined in PLOS consent form) to publish their photographs.

### Experimental procedure

**Fig 1:**
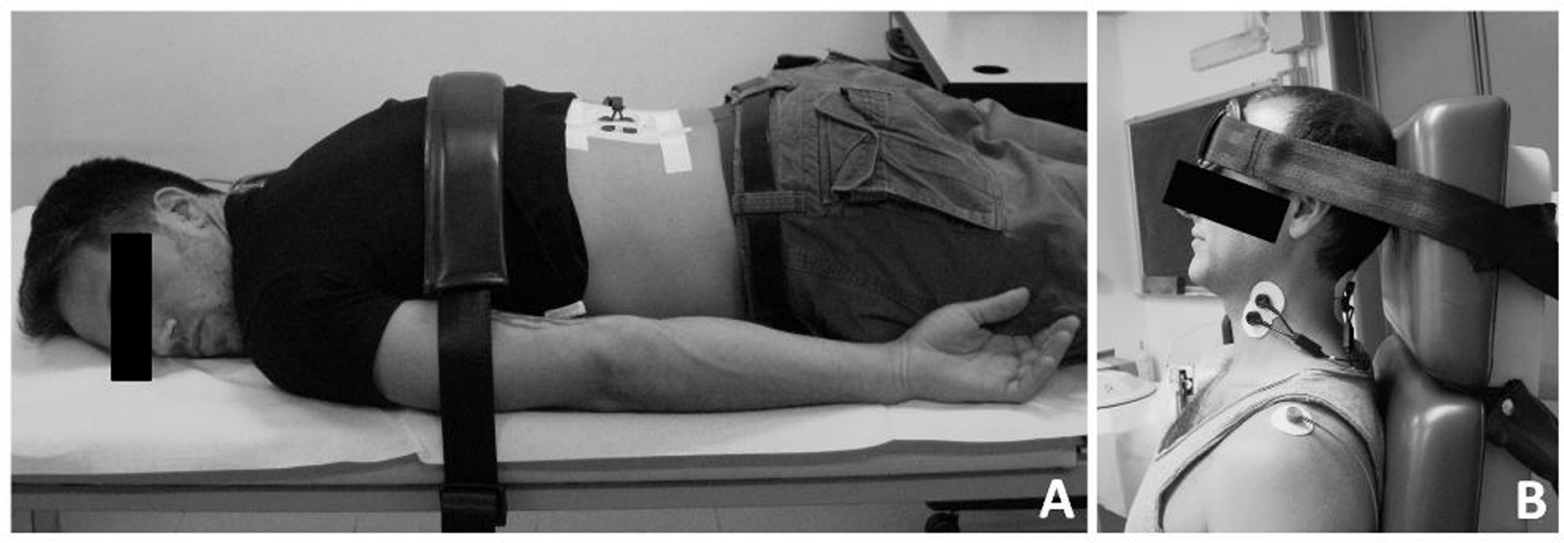
Experimental position for recording maximal voluntary contraction (MVC) of low-back muscles (A) and neck extensor and flexor muscles (B).

Before the driving trials, all subjects were asked to attend the laboratory for the maximal voluntary contraction (MVC) recordings: for the MVC of the back extensor muscles participants were instructed to extend the back while lying in a prone position fastened with a belt (Fig 1, panel A) in order to perform an isometric contraction; similarly, for the MVC of the neck extensor muscles participants were asked to extend the neck against the headrest of a seat. For the MVC of the neck flexor muscles they were asked to flex the neck while the head was stabilized by a belt (Fig 1, panel B). During each MVC participants were verbally encouraged to achieve a maximum and maintain it for at least 2–3 s before relaxing [10,11]. Three MVC attempts were performed, separated by 5 min.

Then subjects were asked to take part in two driving trials by using the same utility car (Wolkswagen Fox) along the same urban circuit, thus covering the same driving distance (15 km). All the experimental sessions were carried out during the summer period (from the 30th of July till the 6^th^ of August) in order to avoid traffic congestion. In each experimental session, all subjects were asked to perform two driving trials: once using the ergonomic devices (Lumbar Support and Neck Balance System DM2; Natura Benessere Salute Srl, Varese, Italy) and once without the ergonomic devices in a random order, with a 30-minutes break period between the trials.

**Fig 2:**
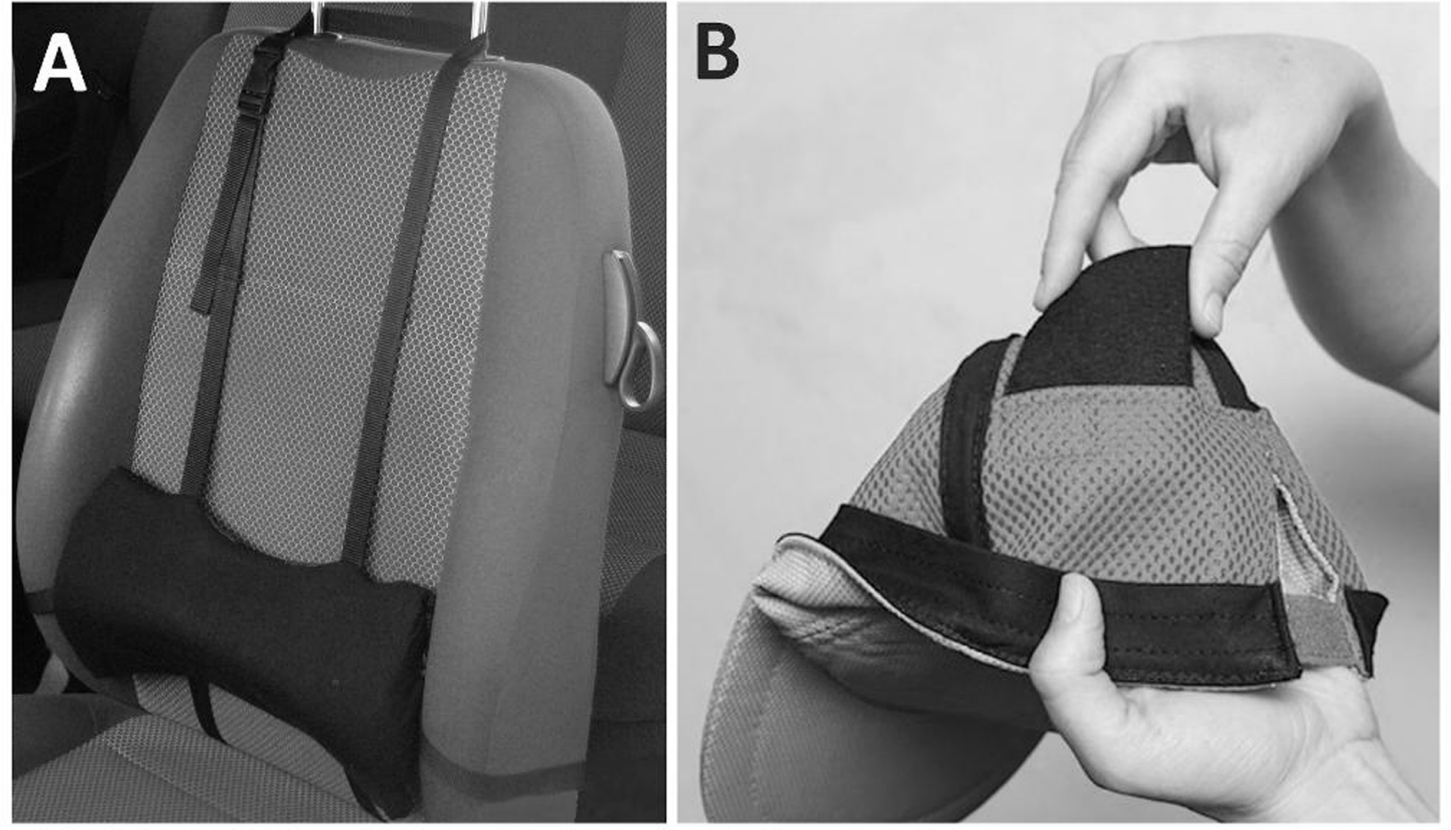
Ergonomic devices: (A) Lumbar Support; (B) Neck Balance System (Natura Benessere Salute Srl, Varese, Italy).

**Fig 3:**
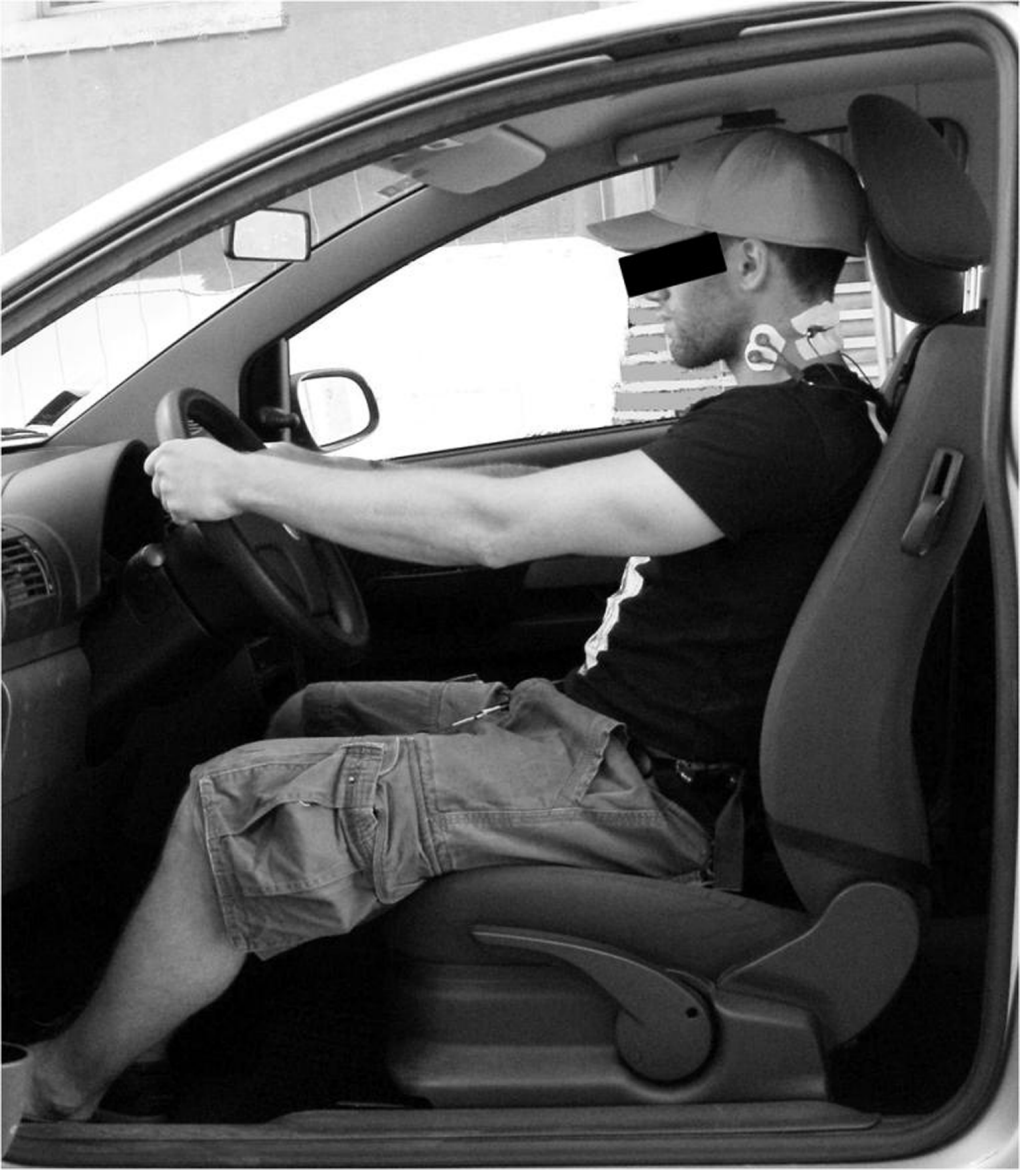
Subject positioning during the driving trials while wearing both ergonomic devices.

The Lumbar Support consists of a polyurethane cushion placed between the vehicle seat and the low back of the subject at L3 level. The positioning of the support was adjusted according to the subjects’ height by regulating two belts, the first fastened to the headrest of the vehicle seat and the second around the back of the seat (Fig 2, panel A). The Neck Balance System is composed by a baseball type cap which contains two weights (0.2 kg each) applied at the occipital level. These two weights are inserted in two appropriate posterior pockets of the device (Fig 2, panel B). An example of the subject’s positioning during the driving trials is shown in Fig 3. Before starting each driving trial, all drivers adjusted their own seats based on personal comfort.

### Instrumentation

Muscle activation was evaluated using surface electromyography (sEMG). SEMG signals were recorded with a portable system (BTS pocket EMG, Milano, Italy) using pre-gelled adhesive electrodes placed in bipolar mode (with a center to center distance of 2 cm). After light skin abrasion and cleaning with alcohol, electrodes were placed bilaterally on the erector spinae (longissimus) of the low back at T10 level, on the semispinalis capitis at C4 level (midway between the occipital protuberance and the bone protuberance of C7) and on the sternocleidomastoid muscle (midway between the mastoid process and the sternal manubrium) according with the SENIAM guidelines. SEMG signals were recorded during both the MVC and driving trials. The sEMG signal was amplified (x1K), low-pass filtered (0 Hz to 500 Hz), and sampled at 1 kHz and stored on a PC laptop at the end of each experimental session for further analyses [12,13].

Mechanical data from the vehicle and the head of the subjects were recorded during the driving trials using two inertial sensors embedding three-axial accelerometers and gyroscopes (3- Space Sensor Data Logging 2.0, Yei technology, Portsmouth, Ohio, USA). One inertial sensor was placed on the dashboard of the car in a horizontal position with a bi-adhesive tape in order to detect acceleration and deceleration phases of the vehicle. A second inertial sensor was placed on the top of the subject’s head, firmly fixed with a Velcro strap on a cap. Signal from the inertial sensors were sampled at 50 Hz, recorded on a micro-SD and stored on a PC laptop at the end of each experimental session for further analysis.

At the beginning of each driving trial a trigger signal was delivered to the sEMG system and inertial sensors for synchronization.

At the end of each driving trial, level of perceived comfort was assessed using a 10-cm visual analogue scale (VAS) [14].

### Data analysis

All data from the sEMG system and the inertial sensors of the driving trials were synchronized and analyzed off-line using Spike 2 6.13 Software (Cambridge Electronic Design, UK). Signals from sEMG were band-pass filtered (10–400 Hz) and the root mean square (RMS) was analyzed over epochs of 250 ms. RMS of the MVC was chosen as the mean value of a 1-s window around the RMS peak of the trial with greatest amplitude. Each of the muscles (sternocleidomastoid muscle, semispinalis capitis muscle, erector spinae muscle), was recorded bilaterally and therefore an average was made between right and left RMS of each muscle.

Signals for the inertial sensors were first analyzed with 3-Space-Sensor Suite 2.0 Software (Yei technology, Portsmouth, Ohio, USA). Variable of interest were the raw antero-posterior accelerations of the vehicle and the pitch of the head. Signals were then low-pass filtered (band pass filter 0–10 Hz) and exported over epochs of 250 ms.

RMS of low-back extensor, neck extensor and neck flexor muscles and the pitch of the head were selected based on the acceleration and deceleration phases of the vehicle. Acceleration phases of the vehicle were identified as the epochs in which the raw antero-posterior accelerations of the vehicle were the positive values above 1 mean SD of all driving trials of all subjects. Similarly, deceleration phases of the vehicle were identified as the epochs in which the raw antero-posterior accelerations of the vehicle were the negative values below 1 mean SD of all driving trials of all subjects. RMS of the neck flexor, neck extensor and back extensor were then expressed as a percentage of the MVC RMS. Head pitch was expressed in degrees as the magnitude of the head rotation around its horizontal axis.

Scores about comfort perception during driving were assessed using a ruler by measuring the distance between zero and the point drown by each volunteer on the 10-cm VAS [14].

### Statistics

All data were normally distributed in terms of skewness and kurtosis (all values less than |2|). A two way ANOVA for repeated measures was carried out to show whether there were any differences in the acceleration and deceleration of the vehicle between trials (with and without device) or between subjects.

Statistical comparisons of variables of interest (low back RMS, neck extensors RMS, neck flexors RMS, head pitch, VAS score) for both vehicle acceleration and deceleration were carried out by paired Student’s t-test. Statistical significance level was set at P<0.05. Unless otherwise specified, data were presented as mean ± standard deviation.

## Results

Acceleration phases of the vehicle, which were identified as the epochs in which the raw antero-posterior accelerations of the vehicle were the positive values above 1 mean SD of all driving trials of all subjects, lasted 167.1 ± 31.5 s with the devices, and 150.2 ± 35.1 s without the devices, with no differences between the two conditions (P>0.05). Deceleration phases of the vehicle, which were identified as the epochs in which the raw antero-posterior accelerations of the vehicle were the negative values below 1 mean SD of all driving trials of all subjects, were 139.7 ± 23.1 s with the devices and 129.3 ± 51.5 s without the devices, with no differences between the two conditions (P>0.05).

The two-way ANOVA for repeated measurements of the vehicle antero-posterior acceleration did not show any significant difference between the two conditions (with devices, without devices) in any subject (P>0.05). Therefore, the lack of differences in vehicle accelerations enabled us to compare muscle activations between trials.

**Fig 4:**
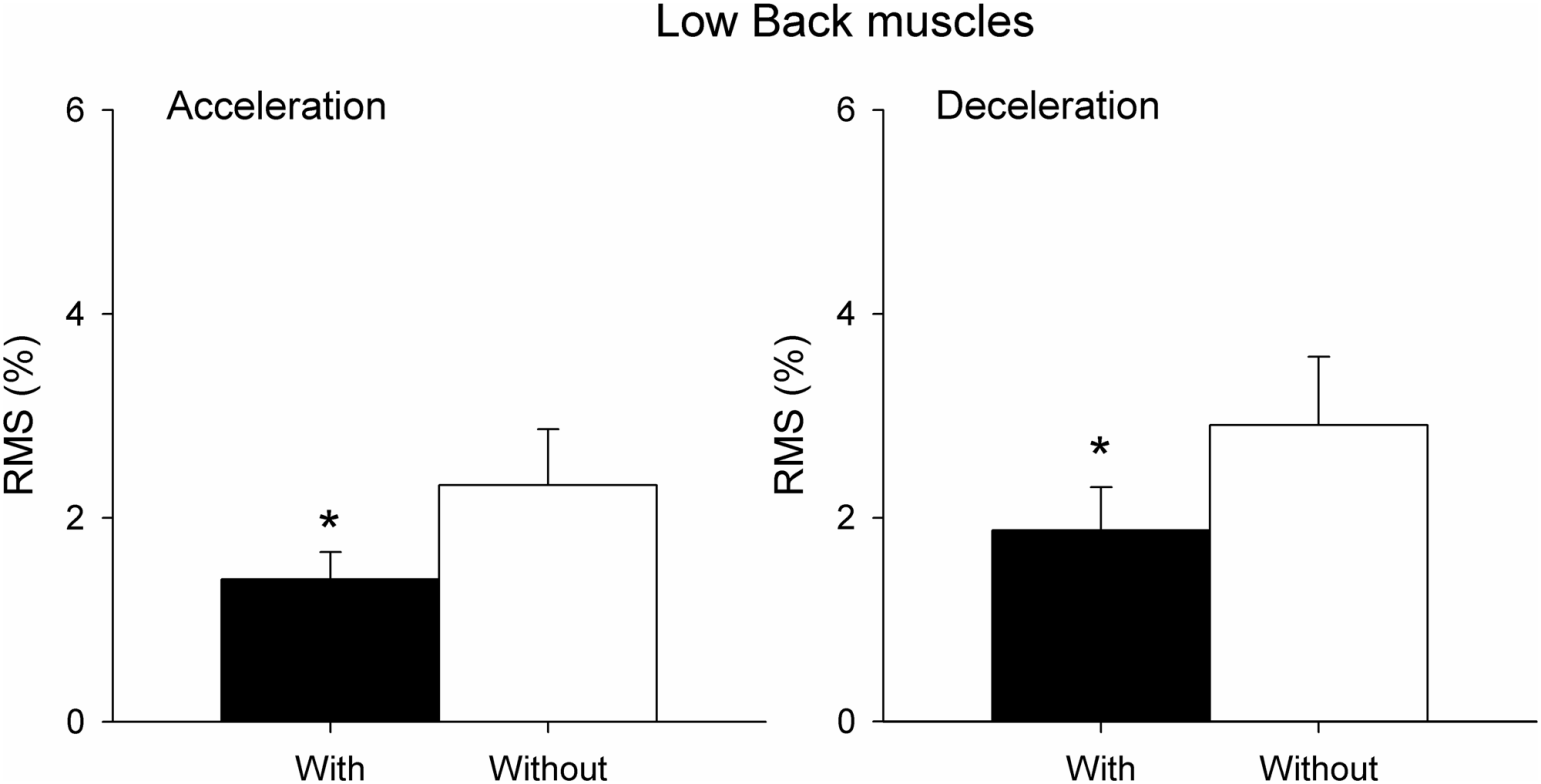
RMS of sEMG of low back muscles (mean±SE) expressed as a percentage of MVC during vehicle acceleration (on the left) and vehicle deceleration (on the right) in participants with and without ergonomic devices. *p <0.05.

RMS of the sEMG signal in the low back muscles for both vehicle acceleration and deceleration phases with and without ergonomic devices are shown in Fig 4. The RMS of the low back muscles was significantly lower when driving with the devices than without in both acceleration phases and deceleration phases of the vehicle (P<0.05).

**Fig 5:**
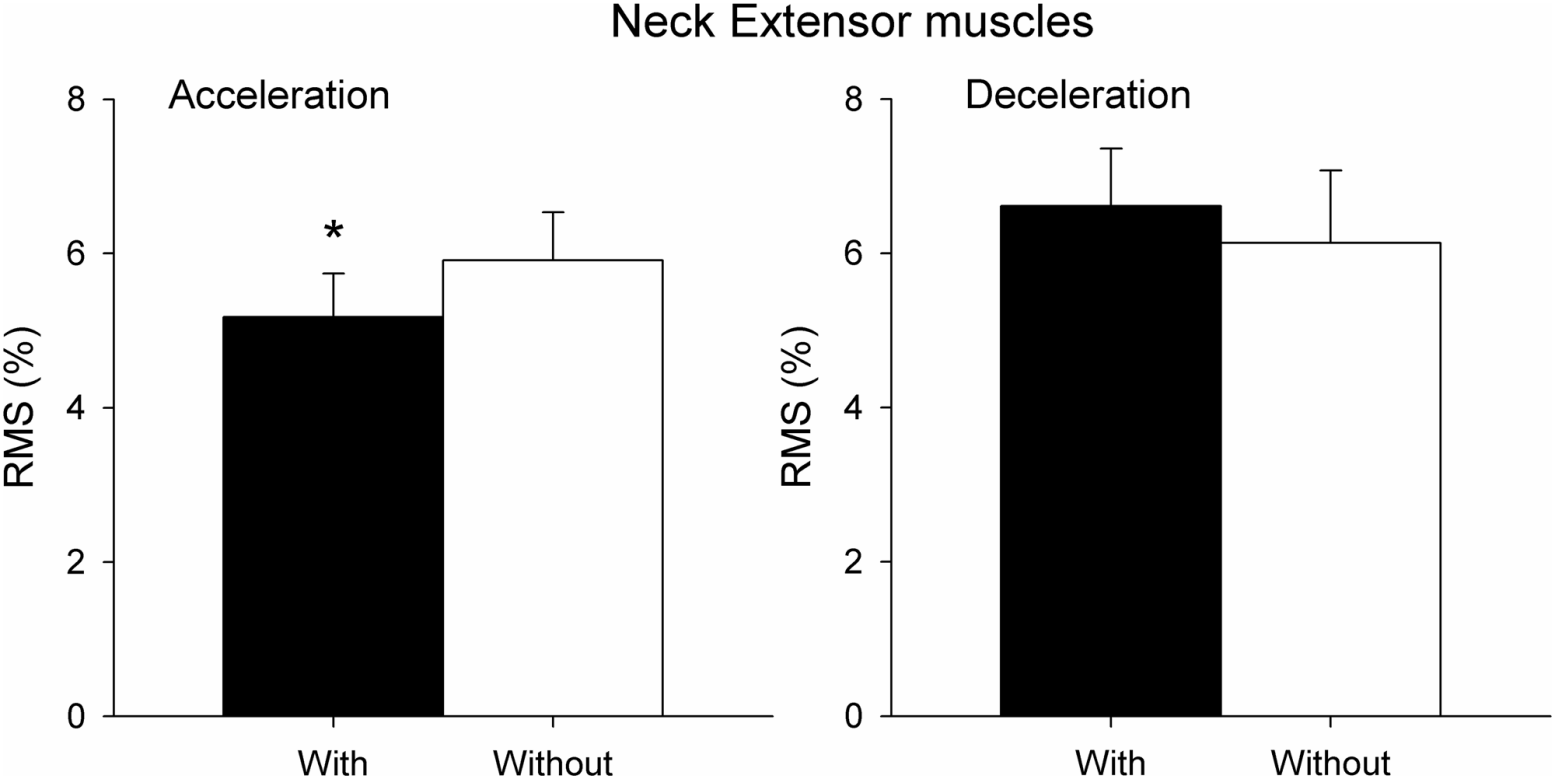
RMS of sEMG of neck extensor muscles (mean±SE) expressed as a percentage of MVC during vehicle acceleration (on the left) and vehicle deceleration (on the right) in participants with and without ergonomic devices. *p <0.05.

RMS of the sEMG signal in the neck extensor muscles for both vehicle acceleration and deceleration phases with and without ergonomic devices is shown in Fig 5. The RMS of the neck extensor muscles was significantly lower when driving with the devices than without during the acceleration phases of the vehicle (P<0.05), but not during deceleration. In contrast, RMS of the sEMG signal in the neck flexor muscles did not differ between the two conditions (with devices, without devices) neither in acceleration phases (2.44%± 1.08 with; 2.34%±1.20 without) nor in deceleration phases (2.11%± 0.91 with; 2.01%±1.02 without; P>0.05).

The pitch of the head did not differ between the two driving conditions (with and without device) during vehicle acceleration (19±4.18 and 17.19±5.92 degrees, respectively) and deceleration (15.77±3.63 and 14.09±3.94 degrees, respectively).

There were no significant differences in comfort perception between driving with ergonomic devices (score 7.48±1.89) and without ergonomic devices (score 7.68± 1.45; P>0.05).

Raw data are available in the supplementary file (S1 Dataset).

## Discussion

The main finding of the present study was that the use of a neck balance system with a lumbar support for both cervical and lumbar rebalance has been shown to reduce activations of both neck extensor and low-back muscles in urban drivers. This could reflect a reduction of the load on cervical and lumbar segments that are caused by the sitting position and the perturbations imposed by the vehicle accelerations and decelerations.

RMS of sEMG signal in the low back muscles was reduced with the use of the two ergonomic devices during driving. This result is in contrast with the findings of Leinonen et al. [7], who did not find any differences in sEMG amplitude of the paraspinal muscles with the use of a low back support during driving. However, the effectiveness of a lumbar support is suggested by the findings of Chen et al. [15], who reported a lower prevalence of low back pain in regularly lumbar support users versus non-users. From early studies on the sitting posture, it is known that using a support at lumbar level leads to a reduction in EMG activity [4]. This observation could be ascribed to a change in lumbo-pelvic angle, as using a lumbar support rotates the pelvis forward and increases the lumbar lordosis [1] towards the normal lumbar curve [16], which is referred to as physiological lumbar curve [17]. Reasonably, when this position is held, the loading on spinal structures is reduced and so postural muscle activations are reduced [4,18].

RMS of sEMG signal in the neck extensor muscles was lower during driving with the two ergonomic devices with respect to driving without the devices, while RMS of the neck flexor muscles did not differ between the two conditions. To the best of the authors’ knowledge, no studies have looked at the acute effect of ergonomic devices for head stabilization on muscular activation during driving. However, our findings complement the results of recent studies on the short- and long-term effects of the neck balance system on neck-head posture and related musculo-skeletal disorders [8,9]. In particular, Pavan et al. [8] reported that, following 15 minutes of cap wearing, the head was retracted, as measured by means of motion analysis. Furthermore, Giombini et al. [9] demonstrated that, after 8 weeks of treatment with Neck Balance System, there was a reduction of neck pain quantified by means of clinical scales. The authors then speculated that the load imposed by the cap could influence postural mechanisms by reducing activation of the extensor muscles which, in turn, would lead to a neck-head posture associated with reduced pain [9]. In addition to the lower activity of the neck extensor muscles, the lack of differences in activity of the neck flexor muscles when driving with ergonomic devices demonstrated that applying a load above the occipital zone, as wearing the cap, did not lead to an extra effort for the neck flexor muscles as a compensatory mechanism. Moreover, evidence of an unchanged neck-head balance comes from the unaltered pitch of the head, which revealed that using or not using the ergonomic devices, did not increase or decrease the oscillation of the head in the sagittal plane following vehicle perturbations (accelerations and decelerations).

There were no differences in the level of perceived comfort assessed by the VAS scale between driving with the two ergonomic devices and driving without the devices. Since there are no studies in the literature reporting the combined effect of two ergonomic devices for cervical and lumbar rebalance, it is not possible to compare our results with observations of others. Nonetheless, it is noteworthy that the use of the ergonomic devices did not alter the level of comfort perceived by the volunteers while driving, which was overall high in both conditions, thus making the use of the device acceptable.

Some limitations need to be addressed. First of all, the experimental design of this study did not allow showing whether or not the combined use of the two devices was superior to using the two devices isolated from each other. Further investigations are needed to explore this issue. A further limitation of the study is that the head pitch was computed by means of YEI Technology proprietary algorithms, which are unknown to the investigators.

In conclusion, the use of a neck balance system together with a lumbar support for both cervical and lumbar rebalance has been demonstrated to reduce the activation of both low-back and neck muscles during driving, which likely reflects a rebalancing of weight on the neck and a neutral position of the lumbar spinal segments, without affecting the level of perceived comfort. A follow-up study will be useful to determine the long-term effectiveness in reducing low-back and neck pain in urban drivers.

## Supporting Information

S1 Dataset Raw data of the experimental study. (XLS)
